# Oral Mucositis Association with Periodontal Status: A Retrospective Analysis of 496 Patients Undergoing Hematopoietic Stem Cell Transplantation

**DOI:** 10.3390/jcm10245790

**Published:** 2021-12-10

**Authors:** Vladimíra Radochová, Martin Šembera, Radovan Slezák, Ondřej Heneberk, Jakub Radocha

**Affiliations:** 1 Department of Dentistry, University Hospital in Hradec Králové, Faculty of Medicine, Charles University, 50003 Hradec Kralove, Czech Republic; martin.sembera@fnhk.cz (M.Š.); slezak@lfhk.cuni.cz (R.S.); ondrej.heneberk@fnhk.cz (O.H.); 2 4th Department of Internal Medicine-Hematology, University Hospital in Hradec Králové, Faculty of Medicine, Charles University, 50003 Hradec Kralove, Czech Republic; radochaj@lfhk.cuni.cz

**Keywords:** oral health, periodontal health, chemotherapy induced oral mucositis, hematopoietic stem cell transplantation

## Abstract

Background: Hematopoietic stem cell transplantation (HSCT) can induce serious oral complications, including oral mucositis (OM). The presence of periodontal inflammation before HSCT is believed to be associated with OM. The aim of our study was to determine the prevalence and severity of OM in patients undergoing HSCT and its relation to periodontal status. Patients and methods: This is a retrospective study of patients who underwent HSCT and a detailed dental examination between 2007 and 2015. The dental and periodontal status of all patients was evaluated by clinical and radiographic examination prior to HSCT. Oral health was assessed with the gingival index, the the community periodontal index, presence of plaque-related gingivitis, and marginal periodontitis. During the HSCT period, patients were examined daily for the presence of OM, which was graded according to World Health Organization (WHO) classification if present. The patients were assigned to the groups according to type of transplantation: autologous HSCT, myeloablative allogeneic HSCT, and non-myeloablative allogeneic HSCT. Results: A total of 496 patients were included in the study. OM was present in 314 of 496 patients (63.3%): 184/251 (73.3%) in the autologous group, 100/151 (66.2%) in the myeloablative allogeneic group, and 30/94 (31.9%) in the nonmyeloablative allogeneic group. Significantly more patients suffered from OM in the autologous and myeloablative groups versus the nonmyeloablative conditioning group (*p* < 0.001). The presence of periodontal inflammation did not significantly differ among the groups. There was only a borderline trend for the higher prevalence of OM in the non-myeloablative allogeneic nonmyeloablative group when periodontal inflammation was present (0.073939). Conclusions: Oral mucositis prevalence and severity after stem cell transplantation is not widely affected by the oral hygiene and periodontal disease presence before HSCT. We confirmed the wide-known connection of the conditioning regimen intensity to the prevalence of OM.

## 1. Introduction

Hematopoietic stem cell transplantation (HSCT) is a widely used procedure. It represents potentially curative treatment for patients with various malignant and non-malignant hematological diseases, including acute and chronic leukemias, aplastic anemia, myelodysplastic syndrome, and lymphomas [1,2]. The conditioning regimen for HSCT results in a dramatic drop in peripheral blood counts, leading to immunodeficiency via neutropenia and agranulocytosis [3]. Chemotherapy and radiotherapy-induced immunosuppression leads to increased susceptibility to infection, including life-threatening septicemia. The oral cavity is a potential source of such infection [1]; therefore, HSCT candidates should undergo a dental examination, and the oral foci of infection should be eradicated before starting the conditioning regimen. Inadequate dental health management may result in HSCT postponement [3]. A number of studies show that a low level of oral health increases the risk of complications in patients after HSCT, including oral mucositis (OM). During neutropenia, oral pathologies become more difficult to treat, and conventional means may not always be possible. The oral foci of infection can exacerbate in local or systemic potentially life-threatening conditions. The inclusion of dentistry in the multidisciplinary context of hemato-oncology is an important part of successful cancer treatment [4,5,6]. On the other hand, some studies have suggested that odontogenic pathologies do not represent a significant risk for systemic infection and dental treatment is unnecessary; moreover, dental treatment may itself result in sepsis [2,7].

OM is defined as inflammation of oral mucosa and represents a major non-hematologic complication of HSCT [8]. It is characterized by toxic damage that affects the entire gastrointestinal tract because of cytotoxic chemotherapy or/and radiotherapy. Nonkeratinized mucosal surfaces, such as the ventral and lateral tongue, floor of the mouth, soft palate, buccal mucosa, and inner sides of the lips, are the most frequently affected. Its biological basis is represented by changes of epithelial atrophy and dyskeratosis with subsequent epithelial breakdown and ulcerations based on observations on animal model [9]. The prevalence of OM varies from 50% to 100% depending on the antineoplastic regimen and patient-associated variables [6,10]. Beyond the direct effect of antineoplastic therapy, additional risk factors include tobacco use, alcohol consumption, young age, female sex, oral foci of infection, poor oral hygiene, poor nutrition, alteration of salivary production, and genetic factors [11]. OM is associated with significant morbidity, pain, odynophagia, dysgeusia, leading to dehydration, malnutrition, and a subsequent reduced quality of life of affected patients. The degree of severity can range from mucosal atrophy, swelling, and mild erythema to severe oral ulcerations. The genesis of OM is described in five overlapping stages: initiation, upregulation and message generation, ulceration, and healing. In neutropenic patients, bacteria may invade into blood flow, causing bacteremia and even sepsis [10]. OM is rarely lethal, but it can lead to increased dosage of antibiotics, opiate analgetics, prolonged hospitalization time, and thus increasing treatment costs. OM typically develops 6–12 days after transplantation and it usually spontaneously resolves in 14–18 days [6]. The aim of the study was to assess the impact of underlying dental and periodontal status on the prevalence and severity of HSCT-induced oral mucositis.

## 2. Materials and Methods

This was a retrospective analysis of patients who underwent HSCT in the 4th Department of Internal Medicine-Hematology, University Hospital in Hradec Králové between 2007 and 2015 and were included in the study. Patient consent was waived due to the retrospective nature of the study. The study was approved by the ethics committee in Hradec Králové University Hospital. Each patient was referred to the Department of Dentistry, Charles University, Faculty of Medicine, and University Hospital in Hradec Králové for examination and elimination of oral foci of infection prior to HSCT. Patients’ details including gender, age, hematologic diseases, HSCT regimen, tobacco abuse, periodontal and dental parameters, development, and severity of OM were obtained from patients’ charts. Patients who refused dental and periodontal examination were excluded from the study. The examination included the plaque index (PI, Silness–Löe Index, 1963) [12], the community periodontal index (CPI) [13], dental caries and teeth vitality. The examination was performed by placing a dental explorer onto the distal part of the tooth surface and drawing it to the medial part on the facial as well as on the oral side of each present tooth. Both the vestibular and oral side of the tooth were separately considered and scored. The criteria for scoring were as follows (PI): 0—no plaque on the tooth surfaces; 1—a film of plaque adhering to the free gingival margin and adjacent area of the tooth. Plaque may be seen in situ only after application of the disclosure solution or by using the probe on the tooth surface; 2—moderate accumulation of soft deposits within the gingival pocket, or the tooth and gingival margin, which can be seen with the naked eye; 3—abundance of soft matter within the gingival pocket and/or on the tooth and gingival margin. The PI score per person was obtained by totaling the scores per tooth surfaces and dividing it by the number of surfaces examined [12]. The CPI was examined with a CPI probe with a 0.5 mm ball tip with a black band between 3.5 and 5.5 mm and rings at 8.5 and 11.5 mm from the ball tip. The mouth was divided into sextants defined by tooth numbers. The criteria for scoring were as follows (CPI): 0—healthy gingiva; 1—bleeding observed; 2—calculus or over hanged fillings and crowns detected during probing, but all the black band on the probe visible; 3—pocket 4–5 mm (gingival margin within the black band on the probe); 4—pocket 6 mm or more (black band on the probe not visible) [13]. Patients were allocated according to periodontal health into following groups: edentulous; healthy periodontal tissues; gingivitis (bleeding upon probing with more than 10% places, no alveolar bone resorption); and periodontitis (bleeding upon probing with more than 30% teeth and alveolar bone resorption). Periapical pathologies were examined though periapical and panoramic radiographs. Patients proceeded to elimination of oral foci of infection. The potentially complicating oral conditions prior to HSCT have been identified as follows: dental caries pulpae proximae, non-vital teeth, apical periodontitis, marginal periodontitis, and semi-impacted teeth. The protocol for the elimination of oral foci of infection elimination consisted of radical teeth extractions. The reasons for extractions were as follows: extraction of teeth with insufficient endodontic treatment quality (extraction of all non-vital teeth in patients undergoing allogeneic HSCT), teeth with periapical lesions, teeth with need of endodontic treatment, advanced periodontal destruction (i.e., the presence of furcation involvement greater than 1st degree, loss of two-thirds of bone support, tooth mobility greater than 1st degree), semi-impacted teeth. Dental treatment following the protocol of oral foci elimination had been managed between 14 and 60 days before the initiation of HSCT. Dental treatment was required to be completed 7 days before HSCT for extraction sites to heal.

Only patients who reached at least partial remission of their respective disease were allowed to proceed to transplantation (exclusive of low-grade myelodysplastic syndrome with less than 5% marrow blasts, aplastic anemia, and myeloproliferative disorders in chronic phase without blast excess). Depending on the type of HSCT and conditioning regimen intensity, they were allocated into three groups: autologous HSCT; allogeneic HSCT myeloablative regimen (MA); and allogeneic HSCT nonmyeloablative regimen (NMA). Each patient experienced at least one episode of severe neutropenia secondary to treatment. ASCT conditioning included Melphalan 200 mg/m^2^ for multiple myeloma and BEAM chemotherapy for lymphomas; both are considered as myelobaltaive. Standard nonmyeloablative conditioning for allogeneic SCT included Busulfan (3.2 mf/kg for 2 days) and Fludarabine (30 mg/m^2^ for 5 days). Standard myeloablative conditioning for allogeneic SCT included Busulfan (3.2 mg/kg for 4 days) plus Fludarabine (40 mg/m^2^ for 4 days) or Cyclophosphamide (60 mg/kg for 2 days) indicated for myeloid malignancies and total body irradiation (12 Gy) plus Fludarabine (40 mg/m^2^ for 4 days) or Cyclophosphamide (60 mg/kg for 2 days) indicated for acute lymphoblastic leukemia. All patients in the alloSCT group received antithymocyte globulin (Thymoglobuline) 3 mg/kg D-2 and D-1 before graft infusion.

Standard anti-infectious prophylaxis during neutropenia for all patients includes ciprofloxacin 2 × 500 mg daily, valaciclovir 2 × 500 mg daily, and fluconazole 400 mg daily (in patients with high risk of invasive fungal infections may be replaced with voriconazole or posaconazole).

Standard graft versus host disease prophylaxis for all allogeneic SCT patients includes tacrolimus 0.03 mg/kg daily (target level 5–15 ng/mL) starting day 1, and mycophenolate mofetil 15 mg/kg twice daily starting D0 after graft infusion.

The patients were closely monitored throughout hospitalization during HSCT; oral examination was performed daily along with a general examination until hospital discharge. The severity of OM was assessed according to World Health Organization (WHO) classification (Table 1). When OM was detected, local anesthetic and anti-inflammatory solutions (preferably chlorhexidine 0.12%) were applied on damaged mucosa. In case of grade 3 and 4 OM, systemic analgesics such as morphine were administered. In case of grade 3, alimentation by sipping was administered. In case of grade 4, total parenteral nutrition or tube feeding was administered.

Tobacco abuse was assessed as follows: non-smoker, 1–10 cigarettes a day, more than 10 cigarettes a day, or quit smoking at least 6 months prior to HSCT.

The chi-square test was performed to determine the relationship between periodontal health (PI, CPI, gingivitis, marginal periodontitis) smoking and OM, as well as the relationship between HSCT type and OM. *p* values of <0.05 were considered statistically significant. Statistical analyses were performed using SPSS Subscription (IBM, New York, NY, USA) and MedCalc v. 9.5.2.0 (MedCalc Software, Ostend, Belgium).

## 3. Results

We identified 520 patients who underwent HSCT; of these, 24 patients declined elimination of dental foci of infection prior to HSCT, thus not meeting the study criteria. Of the 496 patients included in this study, 260 (52.4%) were males and 236 (47.6%) were females. The mean age was 53 years (18–72 years). The most frequent indication for autologous SCT was multiple myeloma (68.1% of patients) and acute myeloid leukemia for allogeneic stem cell transplantation (51.7% for MA conditioning and 34.0% for NMA conditioning). Data concerning gender and HSCT type are summarized in Table 2.

Oral mucositis was recorded in 314 of 496 patients (63.3%): 184 of 251 patients (73.3%) in the autologous group, 100 of 151 patients (66.2%) in the allogeneic MA group and 30 of 94 patients (31.9%) in the allogeneic NMA group. These included oral mucositis grade 1 in 1.6%, grade 2 in 21.9%, grade 3 in 6.7% and grade 4 in 69.8%. Grade 4 mucositis was observed in 129/251 (51.4%) patients after autologous SCT, 71/151 (47.0%) patients after MA allogeneic SCT and 19/94 (20.2%) patients after NMA allogeneic SCT. These differences were statistically significantly different in the NMA versus the MA group (*p* < 0.0001) and the NMA versus the auto group (*p* < 0.0001). However, no differences in the prevalence and/or severity of mucositis were observed in patients with clinically healthy periodontium, gingivitis, periodontitis and edentulous patients (*p* = 0.1792). Regarding smoking, 276 (55.6%) patients were non-smokers, 59 (11.9%) patients smoked 1–10 cigarettes a day, 27 (5.4%) smoked more than 10 cigarettes a day, and 137 (27.0%) quit smoking at least 6 months prior to HSCT. Diabetes mellitus had 41 of 496 patients (8.3%). Gingivitis was present in 223 (45.0%) cases. Marginal periodontitis was present in 240 (48.4%) cases. Statistical analysis showed no significant correlation between smoking and OM (*p* = 0.3642). The impact of periodontal health (PI, CPI, gingivitis, marginal periodontitis) on the occurrence of oral mucositis was not statistically significant (*p* = 0.2601, *p* = 0.7458, *p* = 0.3625, *p* = 0.3933 resp.). The same statistically nonsignificant findings were found in each of the three respective transplantation groups. The prevalence and severity of OM with regard to HSCT type, periodontal status, and smoking is summarized in Table 3, and the specific disease type and oral mucositis is shown in Table 4. Again, no differences were observed in mucositis prevalence and the type of periodontal disease; details are shown in Table 5. We grouped patients according to periodontal disease as patients with periodontal inflammation (periodontitis and plaque-related gingivitis cases) and without (healthy periodontium, edentulous patients), and no differences were observed in terms of mucositis severity; the results are shown in Table 6.

## 4. Discussion

Oral mucositis is one of the most significant complications of both autologous and allogeneic HSCT and causes extended hospitalization, prolonged analgesics including narcotics use, and the incidence of opportunistic infections [14]. The total prevalence of OM during autologous and allogeneic HSCT is 75–100% [15]. A study by Shouval et al. confirmed that the presence of OM prolongs hospitalization and increases the need for intravenous morphine, but on the other hand, there was no increase in the rate of sepsis rate [16]. Rodriguez-Oliveira et al. also demonstrated increased hospitalization costs in patients suffering from OM [17]. Conditioning regimens are the most important parameters determining OM risk [18]. Although the prevalence varies with each study, in the case of grade 2 and more severe OM, the prevalence is 60–67% for autologous HSCT and 30–37% for allogeneic HSCT (non-myeloablative regimen). For allogeneic HSCT (myeloablative regimen), the prevalence of OM is slightly lower than for autologous HSCT [19]. Moreover, OM, following nonmyeloablative regimen, is usually less severe and shorter duration [20]. The prevalence values significantly correlate and are matching our results: autologous HSCT 73.3%, allogeneic HSCT (myeloablative regimen) 66.2%, and allogeneic HSCT 31.9% (*p* < 0.0001). The hematologic diagnosis, which is closely associated with the type of HSCT, was also significantly correlated with the prevalence and severity of OM (*p* = 0.0114).

Several researchers pointed out the oral cavity as a port of entry for the systemic spread of infection in myelosuppressed patients. Bergmann found oral foci in 10.5% of cases of septicemia. In 3.1% of cases, an oral focus of infection was probable or possible [21]. Contrary to a widespread belief, the percentage of documented sepsis is not higher in patients who underwent intensive oral care [22]. Although our results did not show a significant correlation between OM and oral hygiene, the superiority of intensive oral care in both patients with myeloablative and non-myeloablative conditioning regimens has been shown to reduce the risk of mucositis by up to 70% [23]. In addition, Soga performed a retrospective study in leukemia patients and reported that systematic oral care reduced the incidence of oral mucositis by 20% [24]. This was supported by the published study of Kashiwazaki, where the prevalence of OM in patients receiving professional oral health care was 66.7% compared to 93.5% in the group without professional oral health care [25]. Similar results were published by Coracin [26]. Shieh revealed that with proper oral health care support, there was a later onset of OM and a lesser degree of oral mucosal injury [27]. Suwabe et al. also pointed out the decreased incidence of streptococcal bloodstream infections in patients undergoing intensive oral care during HSCT [28]. A study by Kawajiri et al. suggested that oral care could decrease the number of bacteria translocating into the peripheral blood [29]. The intensification of oral care could possibly decrease the prevalence of oral mucositis, as shown by Gaming Legert et al. [30]. Multivariate analysis of risk factors for OM also pointed out the necessity of intensive oral care [31].

It is generally believed that smoking increases the risk of OM. Rugg has proven a highly significant correlation between the prevalence of OM and smoking during and/or after the conditioning regimen (*p* = 0.014) [32]. In our study, there was no significant correlation between the prevalence and severity of OM and smoking (*p* = 0.3642). Interestingly, some studies show that in the presence of chronic irritation and vasoconstriction of smoking on oral tissues, there is a delayed onset of OM with no apparent effect on the incidence or severity of the mucositis. This appears paradoxical, with no simple explanation available [33].

In either group of patients in our study, acute dental and periodontal infections did not occur. This may be because prior to starting chemotherapy, appropriate dental care (including extractions) was administered as necessary in all enrolled patients. Since 1990, dental examination and care have become strongly recommended prior to HSCT [4]. Since then, the necessity of eliminating possible oral foci of infection prior to HSCT is intensely debated. Although most authors agree with this recommendation [1,34,35,36], the main topic remains how radical the protocol should be. However, there are studies concluding that the elimination of oral foci of infection prior to HSCT brings no profit for the patient; on the contrary, it causes new additional problems [2,7].

Another important issue is the actual therapy and prevention of oral mucositis during the transplant procedure. Common preventive strategies include chlorhexidine mouthwash or calcium-enriched mouthwash administered during the transplant procedure. The general efficacy of these approaches in prevention and treatment is low [37]. Palifermin (keratinocyte growth factor) has also failed to show significant efficacy in mucositis management [38]. Therefore, novel approaches are needed. The oral microbiome represents one of the potential targets of therapy [39]. The modulation of local microflora seems to be promising in mucositis therapy as reported by Butera et al. [40]. Another approach using ozonized water has been shown to improve local mucositis in patients with peri-implantitis [41].

## 5. Conclusions

Oral mucositis severity after stem cell transplantation is not widely affected by the oral hygiene and periodontal disease presence before HSCT. Smoking has not increased the risk of OM. We confirmed the wide-known connection of conditioning regimen intensity to prevalence of OM.

## Figures and Tables

**Table 1:** WHO grading or oral mucositis.

Grade	Description
0 (none)	None
I (mild)	Oral soreness, erythema
II (moderate)	Oral erythema, ulcers, solid diet tolerated
III (severe)	Oral ulcers, liquid diet only
IV (life-threatening)	Oral alimentation impossible

**Table 2:** Basic characteristics of the patients.

	Autologous HSCT		Allogeneic HSCT		Allogeneic HSCT		All Patients	
			MA Regimen		NMA Regimen			
	*n* = 251	%	*n* = 151	%	*n* = 94	%	*n* = 496	%
Age (Mean, Range)	57 (21–72)		45 (18–69)		58 (26–70)		53 (18–72)	
Male	141	56.2	76	50.3	53	56.4	270	54.4
Female	110	43.8	75	49.7	41	43.6	226	45.6
Condition								
Acute myeloid leukemia	2	0.8	78	51.7	32	34.0	112	22.6
Acute lymphoblastic leukemia	0	0.0	30	19.9	13	13.8	43	8.7
Chronic lymphocytic leukemia	0	0.0	9	6.0	10	10.6	19	3.8
Chronic myeloid leukemia	0	0.0	5	3.3	3	3.2	8	1.6
Hodgkin lymphoma	15	6.0	1	0.7	0	0.0	16	3.2
Non-Hodgkin lymphoma	58	23.1	9	6.0	8	8.5	75	15.1
Multiple myeloma	171	68.1	0	0.0	3	3.2	174	35.1
Myelodysplastic syndrome	0	0.0	8	5.3	7	7.4	15	3.0
Myeloproliferative disease	2	0.8	8	5.3	13	13.8	23	4.6
Aplastic anemia	0	0.0	2	1.3	1	1.1	3	0.6
Other	3	1.2	1	0.7	4	4.3	8	1.6

Abbreviations: HSCT—hematopoietic stem cell transplantation, MA—myeloablative, NMA—non-myeloablative.

**Table 3:** Prevalence and severity of OM with regard to HSCT type, periodontal status, and smoking.

	Mucositis										
Patient Population	Grade 0		Grade 1		Grade 2		Grade 3		Grade 4		*p* Value
Autologous (*n* = 251)	67	26.7%	3	1.2%	41	16.3%	11	4.4%	129	51.4%	<0.0001
Allogeneic myeloablative (*n* = 151)	51	33.8%	1	0.7%	21	13.9%	7	4.6%	71	47.0%	
Allogeneic nonmyeloablative (*n* = 94)	64	68.1%	1	1.1%	7	7.4%	3	3.2%	19	20.2%	
Periodontal health (*n* = 13)	6	46.2%	0	0.0%	1	7.7%	0	0.0%	6	46.2%	0.1792
Gingivitis (*n* = 214)	71	33.2%	3	1.4%	29	13.6%	7	3.3%	104	48.6%	
Periodontitis (*n* = 189)	64	33.9%	2	1.1%	33	17.5%	9	4.8%	81	42.9%	
Edentulous (*n* = 80)	41	51.3%	0	0.0%	6	7.5%	5	6.3%	28	35.0%	
Non-smokers (*n* = 276)	91	33.0%	3	1.1%	40	14.5%	13	4.7%	129	46.7%	0.3642
Smokers up to 10 cigarettes/day (*n* = 59)	18	30.5%	0	0.0%	8	13.6%	2	3.4%	31	52.5%	
Smokers more than 10 cigarettes/day (*n* = 27)	12	44.4%	0	0.0%	1	3.7%	1	3.7%	13	48.1%	
Former smokers (*n* = 134)	61	45.5%	2	1.5%	20	14.9%	5	3.7%	46	34.3%	

**Table 4:** Oral mucositis in specific disease type.

	Oral Mucositis									
	Grade 0		Grade 1		Grade 2		Grade 3		Grade 4	
	*N*	%	*N*	%	*N*	%	*N*	%	*N*	%
Acute myeloid leukemia	50	44.6	1	0.9	16	14.3	7	6.3	38	33.9
Acute lymphoblastic leukemia	18	41.9	0	0.0	3	7.0	0	0.0	22	51.2
Chronic lymphocytic leukemia	13	68.4	0	0.0	1	5.3	1	5.3	4	21.1
Chronic myeloid leukemia	4	50.0	0	0.0	1	12.5	0	0.0	3	37.5
Hodgkin lymphoma	0	0.0	0	0.0	2	12.5	3	18.8	11	68.8
Non-Hodgkin lymphoma	24	31.6	1	1.3	9	11.8	1	1.3	41	53.9
Multiple myeloma	49	28.0	2	1.1	32	18.3	8	4.6	84	48.0
Myelodysplastic syndrome	7	46.7	1	6.7	3	20.0	0	0.0	4	26.7
Myeloproliferative disease	13	56.5	0	0.0	2	8.7	0	0.0	8	34.8
Aplastic anemia	1	33.3	0	0.0	0	0.0	0	0.0	2	66.7
Other	3	50.0	0	0.0	0	0.0	1	16.7	2	33.3

**Table 5:** Oral mucositis in SCT subgroups according to type of periodontal disease.

	**Mucositis According to Type of Periodontal Disease**										
**Autologous (*n* = 251)**	**Grade 0**		**Grade 1**		**Grade 2**		**Grade 3**		**Grade 4**		***p* Value**
Periodontal health (*n* = 2)	1	50.0%	0	0.0%	0	0.0%	0	0.0%	1	50.0%	0.239
Gingivitis (*n* = 102)	32	31.4%	2	2.0%	15	14.7%	2	2.0%	51	50.0%	
Periodontitis (*n* = 107)	23	21.5%	1	0.9%	23	21.5%	4	3.7%	56	52.3%	
Edentulous (*n* = 40)	11	27.5%	0	0.0%	3	7.5%	5	12.5%	21	52.5%	
	**Mucositis**										
**Allogeneic myeloablative (*n* = 151)**	**Grade 0**		**Grade 1**		**Grade 2**		**Grade 3**		**Grade 4**		***p* Value**
Periodontal health (*n* = 13)	1	14.3%	0	0.0%	1	14.3%	0	0.0%	5	71.4%	0.269
Gingivitis (*n* = 85)	25	29.4%	1	1.2%	12	14.1%	4	4.7%	43	50.6%	
Periodontitis (*n* = 189)	14	33.3%	0	0.0%	6	14.3%	3	7.1%	19	45.2%	
Edentulous (*n* = 17)	11	64.7%	0	0.0%	2	11.8%	0	0.0%	4	23.5%	
	**Mucositis**										
**Allogeneic nonmyeloablative (*n* = 94)**	**Grade 0**		**Grade 1**		**Grade 2**		**Grade 3**		**Grade 4**		***p* Value**
Periodontal health (*n* = 4)	4	100.0%	0	0.0%	0	0.0%	0	0.0%	0	0.0%	0.332
Gingivitis (*n* = 27)	14	51.9%	0	0.0%	2	7.4%	1	3.7%	10	37.0%	
Periodontitis (*n* = 40)	27	67.5%	1	2.5%	4	10.0%	2	5.0%	6	15.0%	
Edentulous (*n* = 23)	19	82.6%	0	0.0%	1	4.3%	0	0.0%	3	13.0%	
	**Mucositis According to Type of Periodontal Disease**										
**Autologous (*n* = 251)**	**Grade 0–2**						**Grade 3–4**				***p* Value**
Periodontal health (*n* = 2)	1			50.0%			1		50.0%		0.569802
Gingivitis (*n* = 102)	49			48.0%			53		52.0%		
Periodontitis (*n* = 107)	47			43.9%			60		56.1%		
Edentulous (*n* = 40)	14			35.0%			26		65.0%		
	**Mucositis According to Type of Periodontal Disease**										
**Allogeneic myeloablative (*n* = 151)**	**Grade 0–2**						**Grade 3–4**				***p* Value**
Periodontal health (*n* = 13)	2			28.6%			5		71.4%		0.073814
Gingivitis (*n* = 85)	38			44.7%			47		55.3%		
Periodontitis (*n* = 189)	20			47.6%			22		52.4%		
Edentulous (*n* = 17)	13			76.5%			4		23.5%		
	**Mucositis According to Type of Periodontal Disease**										
**Allogeneic nonmyeloablative (*n* = 94)**	**Grade 0–2**						**Grade 3–4**				***p* Value**
Periodontal health (*n* = 4)	4			100.0%			0		0.0%		0.229598
Gingivitis (*n* = 27)	16			59.3%			11		40.7%		
Periodontitis (*n* = 40)	32			80.0%			8		20.0%		
Edentulous (*n* = 23)	20			87.0%			3		13.0%		

**Table 6:** Oral mucositis in SCT subgroups according to the presence of periodontal inflammation.

	**Mucositis According to Presence of Periodontal Inflammation**										
**All patients (*n* = 496)**	**Grade 0**		**Grade 1**		**Grade 2**		**Grade 3**		**Grade 4**		***p* Value**
No periodontal inflammation (*n* = 93)	47	50.5%	0	0.0%	7	7.5%	5	5.4%	34	36.6%	0.015
Periodontal inflammation (*n* = 403)	135	33.5%	5	1.2%	62	15.4%	16	4.0%	185	45.9%	
	**Mucositis According to Presence of Periodontal Inflammation**										
**Autologous (*n* = 251)**	**Grade 0**		**Grade 1**		**Grade 2**		**Grade 3**		**Grade 4**		***p* Value**
No periodontal inflammation (*n* = 42)	12	28.6%	0	0.0%	3	7.1%	5	11.9%	22	52.4%	0.0439
Periodontal inflammation (*n* = 209)	55	26.3%	3	1.4%	38	18.2%	6	2.9%	107	51.2%	
	**Mucositis**										
**Allogeneic myeloablative (*n* = 151)**	**Grade 0**		**Grade 1**		**Grade 2**		**Grade 3**		**Grade 4**		***p* Value**
No periodontal inflammation (*n* = 24)	12	50.0%	0	0.0%	3	12.5%	0	0.0%	9	37.5%	0.3636
Periodontal inflammation (*n* = 127)	39	30.7%	1	0.8%	18	14.2%	7	5.5%	62	48.8%	
	**Mucositis**										
**Allogeneic nonmyeloablative (*n* = 94)**	**Grade 0**		**Grade 1**		**Grade 2**		**Grade 3**		**Grade 4**		***p* Value**
No periodontal inflammation (*n* = 27)	23	85.2%	0	0.0%	1	3.7%	0	0.0%	3	11.1%	0.2394
Periodontal inflammation (*n* = 67)	41	61.2%	1	1.5%	6	9.0%	3	4.5%	16	23.9%	
	**Mucositis According to Presence of Periodontal Inflammation**										
**All patients (*n* = 496)**	**Grade 0–2**						**Grade 3–4**				***p* Value**
No periodontal inflammation	54			58.1%			39		41.9%		0.16722
Periodontal inflammation	202			50.1%			201		49.9%		
	**Mucositis According to Presence of Periodontal Inflammation**										
**Autologous (*n* = 251)**	**Grade 0–2**						**Grade 3–4**				***p* Value**
No periodontal inflammation	15			35.7%			27		64.3%		0.223694
Periodontal inflammation	96			45.9%			113		54.1%		
	**Mucositis**										
**Allogeneic myeloablative (*n* = 151)**	**Grade 0–2**						**Grade 3–4**				***p* Value**
No periodontal inflammation	15			62.5%			9		37.5%		0.130235
Periodontal inflammation	58			45.7%			69		54.3%		
	**Mucositis**										
**Allogeneic nonmyeloablative (*n* = 94)**	**Grade 0–2**						**Grade 3–4**				***p* Value**
No periodontal inflammation	24			88.9%			3		11.1%		0.073939
Periodontal inflammation	48			71.6%			19		28.4%		

## Data Availability

The data presented in this study are available on request from the corresponding author.
